# Silent Reading Fluency and Comprehension in Bilingual Children

**DOI:** 10.3389/fpsyg.2016.01265

**Published:** 2016-08-31

**Authors:** Beth A. O'Brien, Sebastian Wallot

**Affiliations:** ^1^Education and Cognitive Development Lab, National Institute of Education, Nanyang Technological UniversitySingapore, Singapore; ^2^Max Planck Institute for Empirical AestheticsFrankfurt, Germany

**Keywords:** text reading fluency, bilingual readers, silent reading, comprehension, recurrence quantification analysis, fractal analysis

## Abstract

This paper focuses on reading fluency by bilingual primary school students, and the relation of text fluency to their reading comprehension. Group differences were examined in a cross-sectional design across the age range when fluency is posed to shift from word-level to text-level. One hundred five bilingual children from primary grades 3, 4, and 5 were assessed for English word reading and decoding fluency, phonological awareness, rapid symbol naming, and oral language proficiency with standardized measures. These skills were correlated with their silent reading fluency on a self-paced story reading task. Text fluency was quantified using non-linear analytic methods: recurrence quantification and fractal analyses. Findings indicate that more fluent text reading appeared by grade 4, similar to monolingual findings, and that different aspects of fluency characterized passage reading performance at different grade levels. Text fluency and oral language proficiency emerged as significant predictors of reading comprehension.

## Introduction

An increasing proportion of individuals worldwide grow up bilingual or multilingual (Grosjean, 2013). Following this, many children learn to read for the first time in what would be a second language (McBride-Chang, 2004). Therefore, it is important to understand what contributes to reading proficiency and comprehension for bi- or multilingual individuals.

The simple view of reading framework (Hoover and Gough, 1990) places reading comprehension as a product of word reading (decoding) and listening comprehension. There is ample evidence from monolingual research to support this view, but more recent findings suggest a role for fluent reading of text which serves as a bridge between word decoding and reading comprehension (Pikulski and Chard, 2005; Adolf et al., 2006; Bashir and Hook, 2009). Reading fluency is found to mediate the relation between reading comprehension and decoding (Silverman et al., 2013), and also to partially mediate the relation between reading comprehension and listening comprehension (Kim and Wagner, 2015).

For second language learners, the simple view of reading is found to hold as it does for monolinguals (Verhoeven and van Leeuwe, 2012), but the role of reading fluency for bilingual or second language readers is not clear. While reading fluency is related to reading comprehension for both monolinguals (Fuchs et al., 2001; Hosp and Fuchs, 2005) as well as L2 readers (Baker and Good, 1995; DeRamirez and Shapiro, 2006), reading fluency research with bilingual children is scarce.

The role of text fluency to reading comprehension for bilingual children requires further investigation for several reasons. First, reading fluency is characterized by automatic word recognition (Samuels, 2006; Kuhn et al., 2010), and developing automaticity for word identification can be a challenge for bilingual readers (e.g., Van Heuven et al., 1998; Segalowitz and Hulstijn, 2005). Bilingual children and adults show slower lexical access (Gollan et al., 2005; Costa et al., 2006; Liu et al., 2010; Sandoval et al., 2010) and smaller receptive vocabularies (Oller et al., 2007; Bialystok et al., 2010) compared with monolingual peers. This may impact on their word fluency. Second, bilingual readers demonstrate poorer reading comprehension that persists despite adequate decoding skills (Proctor et al., 2005; Lesaux et al., 2008; Chen et al., 2012), findings which may be related to poorer text reading fluency found with such readers' performance (Geva et al., 1997; Crosson and Lesaux, 2010; Geva and Farnia, 2012).

Another issue to be resolved is how reading comprehension is related to text fluency *compared with* oral language proficiency in bilingual readers. In most studies, oral reading tasks are used to gauge text fluency, but oral reading may be more heavily influenced by oral language proficiency for bilingual individuals. For instance, lower levels of proficiency may result in slower articulation or mispronunciations during oral reading of a second or additional language. This may result in dysfluent reading (as measured with oral reading rate or prosody), but may not reflect the reader's comprehension of the text (e.g., Geva, 2006). Thus, oral reading fluency may not be an adequate gauge of reading proficiency for L2 or bilingual readers (Piper et al., 2016). For adult bilinguals the relation of oral reading fluency to reading comprehension is found to be weaker for L2 compared with L1 readers, ranging from correlations of 0.26 (Lems, 2012) to 0.46 and 0.51 for L2 adults (Jeon, 2012; Jiang et al., 2012), compared with a correlation of 0.80 for L1 children (Fuchs et al., 2001). With children, oral reading fluency may overestimate bilingual children's comprehension, if their word decoding skills are more advanced than their listening comprehension. Lems (2006) noted that when mastery of decoding skills precedes vocabulary development in L2 readers, they may decode without comprehension, as anecdotally noted by ELL teachers (DeRamirez and Shapiro, 2006). This point was supported by Jackson and Lu's (1992) report of a dissociation between English oral language scores and reading fluency in a group of precocious preschool L2 readers. That is, these children could read as fluently as native speakers did, but had significantly poorer oral language proficiency. Studies with primary school children also showed that oral language proficiency contributes additional variance to comprehension and that it has a moderating effect on the fluency-comprehension relation (Crosson and Lesaux, 2010; Geva and Farnia, 2012).

Thus, fluency measured with a silent reading task should be more independent from oral language proficiency than an oral reading fluency measure. Only a few studies examined the relation of silent reading fluency to comprehension in monolinguals, reporting moderate (*r* = 0.38 in Fuchs et al., 2001) to strong correlations (*r* = 0.75 in Klauda and Guthrie, 2008) for fourth and fifth graders, respectively. There appear to be no studies on the relation of silent reading fluency to comprehension for bilinguals.

Finally, the nature of the fluency-comprehension relation varies developmentally. Reading fluency is expected to transition from word-based to word-integration fluency around age 11 (Berninger et al., 2010), including for L2 reading (Geva and Farnia, 2012). One study with fifth graders suggested that L2 reading fluency may contribute unique variance to reading comprehension beyond word reading fluency and oral language proficiency (Crosson and Lesaux, 2010), supporting a developmental shift to text fluency as a predictor of L2 reading comprehension. Geva and Farnia (2012) similarly found that by Grade 5, word and text fluency formed separate factors for both L1 and L2 readers, and that text fluency contributed uniquely to reading comprehension for both groups.

Around the same period reading shifts from oral to silent mode, which are independent forms of reading (Kim et al., 2011). The use of oral reading for fluency measures, then, may not be appropriate for readers at this age range when reading shifts to silent mode, and this may be particularly true for bilingual readers, as noted above. That is, bilingual readers' oral reading fluency may act simply as a proxy for oral language proficiency in general (e.g., Baker and Good, 1995), and therefore may underestimate children's written language ability. For adult English L2 learners, the correlation of oral and silent reading fluency varies with English proficiency, such that increased proficiency yields a closer correlation between the two reading modes (Lems, 2012).

The focus of the present study is to examine relations between reading fluency and comprehension in bilingual children across the age range where fluency shifts from word- to text-level. To circumvent the above-mentioned issues with oral reading fluency, we measured silent reading fluency of extended text passages in addition to comprehension and word fluency, decoding, and listening comprehension. To appraise silent reading fluency, we applied complexity measures of recurrence and fractal scaling to a self-paced reading task, using word reading times as a series. These complexity measures have been used as a means of quantifying aspects of the reading process, such as stability and structure (Wallot et al., 2012). They are thought to measure the degree to which text and language performance constrain reading (Wallot, 2015), where the better a reader can decode and comprehend a text, the text will have a more systematic influence on reading behavior, as seen in changes in reading process complexity. In previous studies with monolinguals, complexity measures from recurrence quantification and fractal analyses were shown to be a sensitive gauge of individual differences in silent reading fluency. O'Brien et al. (2014) reported that complexity measures of stability and orderliness for reading a text passage varied across age groups with increasing degrees of reading fluency. Wallot et al. (2014) further showed that the complexity measures were better predictors of reading comprehension than reading speed.

In the current study, we examine the relations of fluency and other factors to reading comprehension for bilingual children, across the ages where fluency is expected to shift from word to text level processing. We wanted to examine these relations while keeping comprehension difficulty similar across ages, so we held passage difficulty constant by having children read grade-leveled passages. To investigate what contributes to reading proficiency and comprehension for our bilingual readers, we examined relations between decoding skill, listening comprehension, and fluency with reading comprehension following the simple view of reading. We might expect that decoding skills are relatively less important than listening comprehension within our bilingual sample. Further, we might expect that fluency measured for silent reading additionally contributes to reading comprehension beyond listening comprehension. However, the nature of fluency—comprehension relations may also vary by age, with text fluency becoming more relevant later on. Following these expectations, we address the following research questions:

1. What are the relative contributions of decoding and listening comprehension to reading comprehension within bilingual readers? Does text fluency act as a mediator to reading comprehension?
2. To what extent are text and word fluency related to reading comprehension for bilingual children?
3. Does the relative strength of the fluency-to-reading comprehension relation vary across age groups for word fluency compared with text fluency?

## Methods

### Participants

One hundred and five children in grades 3, 4, and 5 participated (*n*'s = 33, 35 and 37, respectively) from two public schools in Singapore. The children varied in their home language backgrounds, including Mandarin, Malay, Tamil, and others, but all were schooled in English as well as their designated mother tongue. First and primary language use was ascertained with a self-report survey, which included items regarding frequency and domains of use per language, ranking of best language across modes, and family use. A majority of students reported English language (EL) as their best language overall (63), while almost ¼ indicated Chinese (CL) was their best language (24) and 18 reported other languages as their best language [these included Malay (8), Tamil (4) and Burmese, Cantonese, Hokkien, Korean, Myanmar and Tagalog (6 altogether)].

### Measures

All assessments including the story reading task took about 1 h to complete and were given in one session at the child's school.

#### Silent reading fluency for text

Silent reading fluency for text was assessed with an experimental measure of story reading. Each individual read a grade-appropriate story in English that was rendered word-by-word on a MacBook Pro computer using a custom MatLab Psychophysics Toolbox script (Brainard, 1997). As the participant read the story, the words accumulated on the screen in a self-paced manner with a button press for each word (Just et al., 1982). After filling with text, the screen was refreshed for the next page of accumulating text. Response times for each word in the passage were turned into a time-ordered series for submission to non-linear analyses. After reading the story, 10 multiple-choice comprehension questions were read aloud. Questions included literal, inferential, vocabulary from context, and main idea types. There was one story per grade level (based on “Clever Trevor” by Sarah Albee for P3, “Clever Beatrice” by Margaret Willey and Heather Solomon for P4, and “Fiona's Luck” by Teresa Bateman for P5). Stories were modified from published literature to be close to 1100 words long, as necessitated by the non-linear analyses. Details of the story lengths and readability indices are provided in Table 1.

**Table 1:** **Characteristics of story texts**.

**STORY**	**P3**	**P4**	**P5**
Length (words)	1183	1105	1292
Average sentence length (words)	7.2	11.6	11.8
Average word length (letters)	3.9	3.9	4.1
**READABILITY INDECES:**			
ATOS Grade level	2.4	4.4	5.3
Flesch-Kincaid Grade level	1.4	3.0	3.8
**WORD FREQUENCIES:**			
Graded Corpus (WFG)	1427.6	1537.7	1233.2
Total Corpus (WFG)	23062	26236	21108
Singapore Corpus (ICE)	427	382	480

ATOS (Accelerated Reader, 2011); Flesch-Kincaid (Coh-metrix, Graesser et al., 2004); Mean type frequencies according to WFG = Word Frequency Guide (Zeno et al., 1995); ICE = International Corpus of English: Singapore Corpus (Nelson, 2002). Stories correspond to the grade level to which they were admininistered (P3 for grade 3, P4 for grade 4, P5 for grade 5 students).

Complexity measures, were derived using two different non-linear analytic methods: detrended fractal analysis (DFA), multifractal detrended fluctuation analysis (MFDFA), and recurrence quantification analysis (RQA). The appendix to this paper gives a more detailed overview over the three methods, including details of calculating each measure, prior applications, and current interpretations of—and hypothesies regarding—these measures in psychological research.

We used DFA to examine the fractal structure of the series of word reading response times across a text passage. DFA describes how variability changes across different time scales, with a fractal scaling exponent (i.e., *H*, Hurst) that quantifies the degree of long-range correlation in the time series. This scaling exponent, which we refer to in this paper as *monofractal structure*, indicates whether word-by-word response times are independent of each other, or whether there are short-term correlations between response times (e.g., reading word_n_ affects the reading of adjacent word_n+1_) or perhaps longer-term correlations across larger segments of the text (e.g., words within sentences or paragraphs or whole passages). From previous work (Kloos and Van Orden, 2010; Kuznetsov and Wallot, 2011; O'Brien et al., 2014; Wallot et al., 2014), we conceptualize that more proficient reading is guided and constrained by extraneous text features and therefore exhibits weaker long-term traces or links across word reading times during self-paced reading (O'Brien et al., 2014; Wallot et al., 2014) or fixation duration during reading (Wallot et al., 2015). Essentially, this means that more proficient, fluent reading is characterized by reduced scaling, with *H* relatively closer to random fluctuations or white noise.

A second variable we estimated is *multifractal structure*, using MFDFA, which is an expansion of DFA. Multifractal scaling captures the degree to which monofractal structure changes in the response-time series. Multifractal structure signifies that the series of reading times is heterogenous and exhibits interactions across time-scales (Ihlen and Vereijken, 2010; Kelty-Stephen et al., 2013), for example where different levels of discourse (topical, syntactical, semantical, sub-lexical) interact with each other to guide readers' comprehension (Booth et al., 2016). It is expected that multifractal structure may capture a reader's adaptive behavior while reading a text for comprehension (Wallot et al., 2014). In this case, sudden on-line changes may occur during reading, perhaps reflecting a more dramatic change in understanding or insight (Stephen et al., 2009), rather than more gradual shifts where meaning is built cumulatively from preceding text (Donald, 2007).

The third non-linear method, RQA, quantifies recurrent patterns in the reading time series, describing the system's stability or orderliness. This analysis yields estimated parameters of the orderliness or recurrent patterning of a system within the task's phase space, quantified as the proportion of data points that are part of a recurring pattern, and referred to here as *%Determinism.* Prior studies show that reading performance becomes more structured with higher determinism as reading skill increases (Wijnants et al., 2009, 2012; Wallot et al., 2012), and determinism is higher in more fluent readers (O'Brien et al., 2014; Wallot et al., 2014). It has been suggested that measures of temporal structure of reading times, such as RQA %Determinism, capture how well readers utilize the informational structure of a text, and conversely, how well a texts constrains the reading process toward efficient and fluent reading (Wallot, 2015, 2016). Hence, high degrees of %Determinism should be positively correlated with aspects of reader skill. In that sense, *monofractal structure* and *%Determinism* are conceptually closely related, but there are differences as well (see Appendix).

Thus, for our analysis we include three complexity measures to capture aspects of structure and orderliness of reading times over the series of the text: monofractal structure, multifractal structure, and %Determinism.

#### Word reading fluency

Word reading fluency was assessed with the TOWRE (Test of Word Reading Efficiency) sight word subtest (Torgesen et al., 1999). This test involves sight word reading of a list of words, starting from more to less frequent. Scores are tallied as number of correctly read words within the 45 s time limit.

#### Decoding

Decoding was assessed with the TOWRE phonemic decoding subtests (Torgesen et al., 1999). Similar to the above subtest, this test involves reading of a list of non-words by phonemically decoding them, and scores are tallied as the number of correctly read non-words within the 45 s time limit.

#### Reading component skills

Reading component skills including phonological awareness and rapid symbol naming, two robust predictors of reading ability, were also assessed and were used descriptively. *Phonological awareness* was measured with the CTOPP (Comprehensive Test of Phonological Processing) Elision subtest (Wagner et al., 1999) which is a phoneme deletion task. *Rapid symbol naming* was assessed with the RAN/RAS (Rapid Automatized Naming and Rapid Alternating Stimulus Tests) letters subtest (Wolf and Denckla, 2004).

#### Oral language proficiency

Oral language proficiency was assessed with the WJIII (Woodcock-Johnson III) Listening Comprehension Cluster, which is comprised of the WJIII-Understanding Directions and Oral Comprehension subtests (Woodcock et al., 2007). Understanding directions involves listening to increasingly complex sequences of instructions and responding by pointing to objects in a picture. Oral comprehension involves listening to short passages and using semantic/syntactic cues to supply missing words within the passage.

### Data preparation

Prior to FA, extreme response times of 10 s or longer were removed, a threshold adapted to children's reading times (O'Brien et al., 2014). On average, 3.7 data points were eliminated per participant (*SD* = 4.0), which amounted to 0.31% of all data points. The extreme scores were removed because they can distort the fractal analysis, while the slight disruption on the series' time order has minimal impact, as long as a minimum of 1024 observations are maintained (Holden, 2005, p. 285–287). There are several methods available to estimate the scaling relations, including spectral analysis (SA), standardized dispersion analysis (SDA), and detrended fluctuation analysis (DFA). DFA results are reported here (Peng et al., 1995), and were corroborated with the two other methods of FA. To assess multifractal structure, mulifractal detrended fluctuation analysis (MFDFA) was used (Kantelhardt et al., 2002).

For RQA, all data in the time-series were entered into the analysis using the Commandline Recurrence Plots software (Marwan, 2011). Data are first rescaled relative to the Euclidian distance separating points in reconstructed phase space (using time-delayed copies of the time series as surrogate dimensions) providing an intrinsically scaled metric across the set of data. %Determinism was calculated using parameters of embedding delay (1), dimension (5) and radius (0.4) following procedures described in Webber and Zbilut (2005).

For the standardized assessments of word reading, decoding, phonological awareness, rapid naming, and listening comprehension Student's *t*-statistic was used for the correlation and multiple regression analyses given the difference in the current sample from the normative sample.

## Results

### Descriptives of reading skills

Average performance per grade level on the standard reading and language measures are shown in Table 2, along with the overall sample mean. Standard scores are presented here, to give an impression of peer-referenced skill levels across grades, but it should be noted that the standardized scores are based on published normative data from monolingual English speakers. As can be seen most of the averages are within the normal range, with the exception of Listening Comprehension, which is about 1 SD or more below the mean for P3 and P4 groups.

**Table 2:** **Mean (SE) standard scores on reading and language tests**.

**Test**	**Grade 3 (*n* = 33)**	**Grade 4 (*n* = 34)**	**Grade 5 (*n* = 37)**	**Overall (*N* = 105)**
**READING COMPONENT SKILLS**				
Rapid Naming	111.4 (2.6)	110.0 (2.8)	118.7 (2.3)	113.5 (1.5)
Phonological awareness	8.3 (0.3)	8.0 (0.5)	8.9 (0.5)	8.4 (0.2)
Decoding	104.8 (2.4)	107.1 (2.4)	111.2 (1.6)	107.8 (1.2)
Word reading fluency	111.9 (2.1)	110.0 (1.9)	93.6 (2.1)	111.7 (1.1)
Listening Comprehension	81.9 (2.7)	82.9 (2.6)	89.3 (2.4)	84.8 (1.5)

Rapid Naming (RAN/RAS), Decoding (TOWRE-phonemic decoding), Word reading fluency (TOWRE-sight words), and Listening comprehension (WJIII) standardized scores are relative to a mean of 100 (SD = 15). Phonological awareness (CTOPP-Elision) is a scale score with a mean of 10 (SD = 3).

Performance on the silent passage reading task is presented in Table 3 for reading rate (wpm) and comprehension scores and the complexity measures. Notably text comprehension did not differ across the three grade level groups [*F*_(2, 101)_ = 1.70, *p* = 0.189, η^2^ = 0.032], most likely as a result of matching the text difficulty appropriately for each grade. Reading speed, on the other hand, did increase significantly across the grades [*F*_(2, 101)_ = 9.72, *p* < 0.001, η^2^ = 0.161], with 3rd graders reading slower than 4th and 5th graders (*p'*s < 0.001), but no difference for 4th and 5th graders in reading speed (*p* = 0.783) according to Bonferroni corrected *post-hoc* tests.

**Table 3:** **Performance measures on the silent passage reading task**.

**Test**	**Grade 3 (*n* = 33)**	**Grade 4 (*n* = 34)**	**Grade 5 (*n* = 37)**	**Overall (*N* = 105)**
Reading Rate (WMP)	100.2 (32.6)	132.3 (50.4)	134.8 (42.1)	123.0 (45.0)
Story Comprehension	68% (20–100)	76% (20–100)	77% (30–100)	74% (20–100)
**COMPLEXITY MEASURES**				
%Determinism	0.86	0.92	0.93	0.90
Monofractal Structure	0.66	0.49	0.44	0.52
Multifractal Structure	0.76	0.97	0.85	0.86

Silent reading rate is reported in words per minute, and Story comprehension is percent correct, with the group's range shown below.

**Figure 1:**
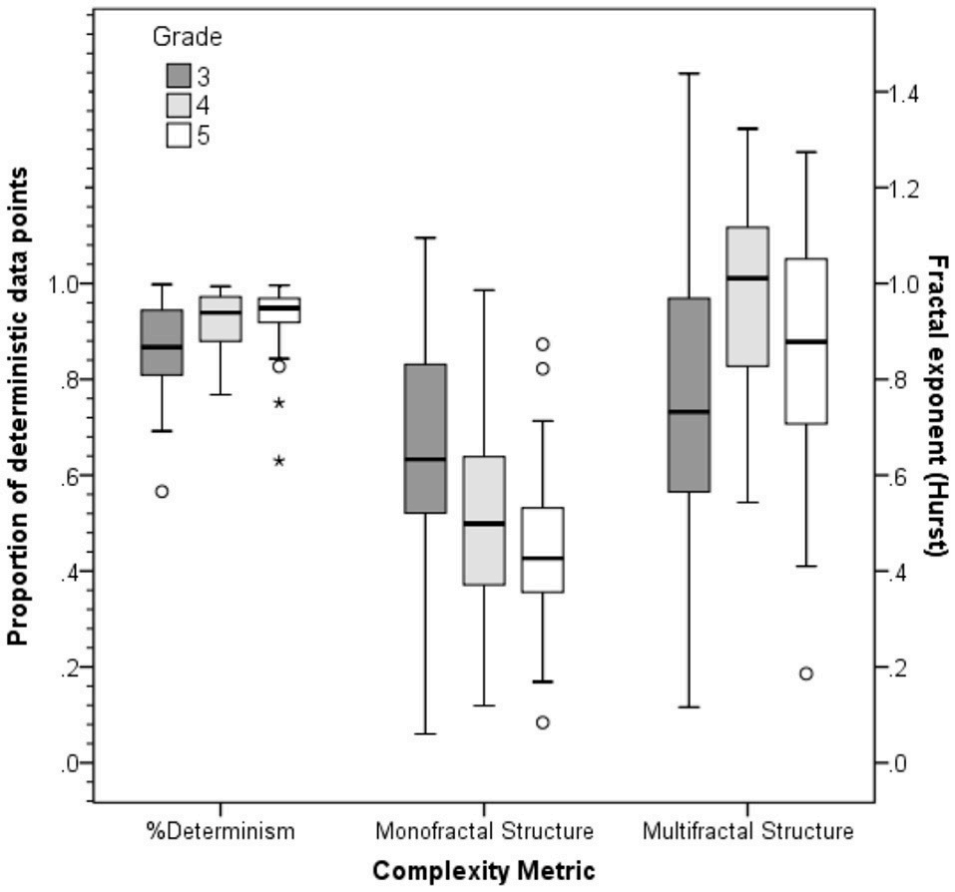
**Complexity metrics of silent reading fluency for text**. Performance by grade-level groups on %Determinism, monofractal structure, and multifractal structure measures of silent story reading. Complexity metrics are computed from the Recurrence Quantification (RQA) and Fractal Analyses (FA) of individuals' series of word reading response times across the text passage. Determinism from RQA is reported in percent of recurrent points, and monofractal and multifractal structure are reported as Hurst exponents. ^*^indicates outliers.

For the complexity measures applied to the text reading times series, there was a significant increase in *%Determinism* of reading times across grades [*F*_(2, 101)_ = 7.63, *p* < 0.001, η^2^ = 0.131], indicating that word reading times became more regular for older readers. Bonferroni corrected *post-hoc* tests revealed that 3rd graders showed lower %Determinism of reading times than 4th (*p* = 0.003) and 5th graders (*p* = 0.002), but 4th and 5th graders did not differ in %Determinism (*p* = 0.929). *Monofractal structure* in reading times also differed across grades [*F*_(2, 101)_ = 10.21, *p* < 0.001, η^2^ = 0.168]. *Post-hoc* tests with Bonferroni correction showed that the 3rd grade group had a greater fractal exponent than both 4th (*p* = 0.003) and 5th graders (*p* < 0.001). This differs from the previous finding with monolingual children, who showed no age effects of monofractal structure (O'Brien et al., 2014). Furthermore, we analyzed the change of *multifractal structure* in reading times, which increased with grade *F*_(2, 101)_ = 5.65, *p* = 0.005, η^2^ = 0.101. Bonferroni corrected *post-hoc* tests revealed that 3rd graders showed less multifractal structure in reading times compared to 4th graders (*p* = 0.004). No other effects were apparent (both *p* > 0.133). Figure 1 shows the group means for each of the three complexity metrics.

To examine relations across all measures, including traditional literacy and language proficiency tests as well as the silent reading task, we calculated zero-order Pearson-correlations with age partialled out. For the standardized tests (CTOPP, TOWRE, WJIII) we used Student's t-statistic based on the sample's mean and standard deviation. From Table 4, it appears that rapid naming had a stronger relation to decoding and word fluency skills than phonological awareness, and a small correlation with monofractal structure. Phonological awareness was not systematically related to the complexity measures. Decoding and word fluency skills, on the other hand, were related to each other, and showed similar relations with monofractal structure and listening comprehension, but only word fluency was correlated with reading comprehension. Further, while the complexity measures for silent text reading showed some interrelationships, it is only monofractal structure that showed a relation to reading comprehension scores. This correlation was stronger than that between word fluency and reading comprehension. Listening comprehension showed strongest correlation with reading comprehension for this sample.

**Table 4:** **Correlations between reading and language measures for the whole sample**.

	**2**.	**3**.	**4**.	**5**.	**6**.	**7**.	**8**.	**9**.
1. Rapid Naming	0.019	−0.**368**	−**0.675**	−0.166	0.304^*^	−0.073	−0.136	−0.169
2. Phonological awareness	1	0.247^*^	0.043	−0.013	−0.076	−0.158	0.101	−0.014
3. Decoding		1	**0.577**	0.220^*^	−**0.384**	−0.056	**0.274**	0.154
4. Word reading fluency			1	0.231^*^	−**0.417**	0.085	**0.296**	0.259^*^
5. %Determinism				1	−**0.345**	**0.472**	**0.271**	0.037
6. Monofractal structure					1	0.128	−**0.326**	−**0.310**
7. Multifractal structure						1	0.025	0.004
8. Listening comprehension							1	**0.534**
9. Reading Comprehension								1

Age Partialled Out.

* marks p < 0.05; bold marks p < 0.01.

Measures include Student's t-statistic for rapid naming (RAN letters subtest), phonological awareness (CTOPP Elision subtest), decoding efficiency (TOWRE phonemic decoding subtest), and word reading fluency (TOWRE sight word subtest), and listening comprehension (WJIII), and percent correct for reading comprehension. Complexity measures for silent reading fluency include %Determinism, and mono- and multifractal scaling exponents.

### Relation of components of the simple view of reading

To address the first research question hierarchical regression models were run with reading comprehension as the criterion measure. Age was entered as the first step, then decoding was entered into the second step and listening comprehension scores into the final step. Overall the model accounted for 29% variance in reading comprehension (see Table 5). Decoding did not contribute significantly, but listening comprehension did, accounting for 25% unique variance in reading comprehension after accounting for the other variables. When the order of decoding and listening comprehension predictors was reversed, listening comprehension still accounted for significant variance and decoding did not contribute any additional variance. This confirms the first hypothesis that listening comprehension would be a more potent factor for reading comprehension compared with decoding in our bilingual sample.

**Table 5:** **Hierarchical Multiple regression predicting reading comprehension from decoding and listening comprehension**.

**Step**	**Variables entered**	**Δ*R*^2^**	**β**	***p***	**Variables entered**	**Δ*R*^2^**	**β**	***p***
1	Age	0.021	0.146	0.14	Age	0.012	0.146	0.14
2	Age	0.023	0.153	0.12	Age	**0.287**	0.164	0.05
	Decoding		0.153	0.12	Listening comprehension		0.529	0.00^*^
3	Age	**0.256**	0.165	0.05	Age	**0.280**	0.165	0.05
	Decoding		0.008	0.93	Listening comprehension		0.527	0.00^*^
	Listening comprehension		0.527	0.00^*^	Decoding		0.008	0.93

Bold indicates significant change in explained variance. Listening comprehension (WJIII) and Decoding (TOWRE), predictors entered as Student's t-statistic.

* indicates significant effect of predictor.

To test the second prediction that fluency plays a mediating role for reading comprehension and either decoding or listening comprehension, mediation analysis was run using structural equation modeling (Lavaan statistics package within R, Rosseel, 2012). First, a model of reading comprehension scores with decoding as the predictor and one of the text fluency measures as mediator was run. Only the model with monofractal structure entered as mediator showed a significant indirect effect (indirect effect *Z* = 2.49, *p* = 0.013, direct effect *Z* = 0.33, *p* = 0.74, *R*^2^ = 0.115). The models with %Determinism and multifractal structure showed no significant effects, either direct or indirect, of decoding on reading comprehension (*R*^2^'s = 0.02). Second, models of reading comprehension regressed on listening comprehension revealed significant direct effects with %Determinism or multifractal structure as a mediator (*Z* = 6.3, *p*'s < 0.01, *R*^2^'s = 0.27). Only monofractal structure showed a trend toward a significant mediation effect for the listening and reading comprehension relation (indirect effect *Z* = 1.89, *p* = 0.059, direct effect *Z* = 5.45, *p* < 0.01, *R*^2^ = 0.311).

Thus, in this current bilingual sample, the measure of decoding skill was a much weaker predictor of reading comprehension than the measure of listening comprehension skill, and only showed an indirect effect on reading comprehension through text fluency (monofractal structure). Listening comprehension, on the other hand, was directly related to reading comprehension, and only monofractal structure for text fluency showed a tendency to mediate this relation.

### Relation of word level and text level fluency to reading comprehension

To address the second research question hierarchical regression models were run with reading comprehension as the criterion measure and fluency measures as predictors. Age was entered as the first step, then word reading fluency was entered into the second step. The three complexity measures (%Determinism, Monofractal structure, and Multifractal structure) were entered into the last step of the model. Overall the model accounted for almost 14% variance in reading comprehension (see Table 6). The model with word fluency and age tended toward significance (*p* = 0.06), whereas the addition of the text fluency variables showed a significant change in explained variance for the model. Of the three text fluency measures, monofractal structure accounted for 8% unique variance, while contribution of variance from the other complexity measures was not significant. For the reverse order of entry, with text fluency measures entered in the second step and word fluency in the final step, text fluency, and age accounted for 11% of the variance in comprehension, and word fluency did not add significant variance beyond this.

**Table 6:** **Hierarchical Multiple regression predicting reading comprehension from reading fluency measures**.

**Step**	**Variables entered**	**Δ*R*^2^**	**β**	***p***	**Variables entered**	**Δ*R*^2^**	**β**	***p***
1	Age	0.021	0.146	0.14	Age	0.021	0.146	0.14
2	Age	0.035	0.160	0.10	Age	**0.110**	0.034	0.74
	Word fluency		0.186	0.06	%Determinism		−0.156	0.21
					Monofractal		−0.397	0.00^*^
					Multifractal		0.125	0.28
3	Age	**0.080**	0.053	0.62	Age	0.005	0.053	0.62
	Word fluency		0.077	0.47	%Determinism		−0.167	0.19
					Monofractal		−0.365	0.00^*^
					Multifractal		0.126	0.28
	%Determinism		−0.167	0.19	Word fluency		0.077	0.47
	Monofractal		−0.365	0.00				
	Multifractal		0.126	0.28				

Bold indicates significant change in explained variance. Listening comprehension (WJIII) and Decoding (TOWRE), predictors entered as Student's t-statistic.

* indicates significant effect of predictor.

### Word and text fluency across grade

Next we addressed the third research question, and the prediction that fluency shifts from word level to text level around fourth grade. It was of interest to see whether there were different patterns amongst these measures at any juncture across the hypothesized developmental shift from word- to text-reading. Intercorrelations for each grade are shown separately in Tables 7–9. As predicted, the influence of word reading fluency on reading comprehension declined with age, and was only significantly correlated in the third grade group. The measures of text fluency, on the other hand, showed different patterns of variation over the three age groups. Monofractal structure, like word fluency, was correlated with reading comprehension only for the P3 group, showing no significant relation for fourth and fifth grade children. Multifractal structure and %Determinism showed the opposite pattern, whereby they were not significantly related to reading comprehension in grade 3 or 4 groups, but were related in the fifth grade children.

**Table 7:** **Relation between reading fluency measures and reading comprehension for Grade 3**.

	**2**.	**3**.	**4**.	**5**.
1. Word Fluency	0.415^*^	−**0.621**	−0.063	**0.535**
2. %Determinism	1	−0.406^*^	0.431^*^	−0.128
3. Monofractal		1	0.259	−**0.510**
4. Multifractal			1	−0.299
5. Reading Comprehension				1

* marks p < 0.05; bold marks p < 0.01.

**Table 8:** **Relation between reading fluency measures and reading comprehension for Grade 4**.

	**2**.	**3**.	**4**.	**5**.
1. Word Fluency	0.367^*^	−0.346^*^	0.190	0.019
2. %Determinism	1	−0.296	0.279	−0.095
3. Monofractal		1	0.072	−0.098
4. Multifractal			1	−0.038
5. Reading Comprehension				1

* marks p < 0.05.

**Table 9:** **Relation between reading fluency measures and reading comprehension for Grade 5**.

	**2**.	**3**.	**4**.	**5**.
1. Word Fluency	−0.102	−0.246	0.154	0.189
2. %Determinism	1	−0.109	**0.602**	0.335^*^
3. Monofractal		1	0.148	−0.186
4. Multifractal			1	0.398^*^
5. Reading Comprehension				1

* marks p < 0.05; bold marks p < 0.01.

Finally, we regressed reading comprehension on the fluency measures using stepwise multiple regression with a forward selection procedure. This allowed us to examine which of the fluency measures improved the prediction model best within each grade, although results are viewed with caution because the sample size per grade was small. The regressions confirmed the above observations from the correlation tables. For grade 3, significant predictors of reading comprehension included both word fluency (semipartial correlations, *r* = 0.373) and the text fluency measures of monofractal structure (*r* = −0.313) as well as %Determinism (*r* = −0.442) (*R*^2^ = 0.485, *F* = 6.1, *p* = 0.02). For grade 4, none of the fluency measures contributed significantly to the prediction of reading comprehension, whereas for grade 5 only multifractal structure of text fluency was a significant predictor (*r* = 0.398, *R*^2^ = 0.134, *F* = 6.4, *p* = 0.016). Thus, there was an overall shift from word to text fluency over these grade levels.

## Discussion

The current study examined the relation between silent reading fluency and comprehension for bilingual children across the age range where fluency is proposed to shift from word-level to text-level. While some of the findings replicate those with monolingual readers, there were also some differences with regard to the interrelations of skills and processes with reading comprehension, and to age-related variations in fluency of silent text reading.

Interrelations between the fluency measures and basic reading related skills showed that rapid naming, but not phonological awareness, was related to both word fluency and silent text reading fluency. This follows from prior monolingual research where rapid naming is a better predictor of word fluency, and phonological awareness is better for predicting word reading accuracy (e.g., Schatschneider et al., 2004). Interestingly, rapid naming was related to the text fluency metric of monofractal structure, which, unlike word fluency, differs from RAN in that it is not a simple rate measure, but an indicator of the structure of reading times across the text.

The text fluency metrics of %Determinism and monofractal structure were also related to skills of decoding and word fluency, and all four of these measures were correlated with listening comprehension. Decoding and listening comprehension tend to show stronger correlations within monolingual samples (*r*'s = 0.40 to 0.60, Foorman et al., 2015) compared with here (*r* = 0.27), implying that these skills may be more dissociated or develop more independently in bilingual readers (e.g., Jackson and Lu, 1992).

Reading comprehension, on the other hand, was not significantly related to decoding, in contrast to findings with monolingual readers of similar age (Foorman et al., 2015). Only listening comprehension, along with word fluency and the text fluency measure of monofractal structure, were significantly correlated with reading comprehension. Listening comprehension was a significant predictor of reading comphrension, explaining 25% unique variance beyond age and decoding skills, and showing a direct effect on reading comprehension. Decoding skills did not show such an impact, but was only related to reading comprehension indirectly, mediated by text fluency (monofractal structure). This further supports the prediction that decoding would play a lesser role in reading comprehension for bilingual readers. The findings also support the role of fluency as a mediator between decoding and comprehension, similar to findings with monolingual readers (Silverman et al., 2013), and suggests that issues with poor comprehension are more related to fluency than decoding for bilingual readers (e.g., Crosson and Lesaux, 2010; Chen et al., 2012; Geva and Farnia, 2012).

Language skills are found to relate to text fluency for monolingual readers (Cutting et al., 2009), and even moreso for second language learners (Geva and Zadeh, 2006; Crosson and Lesaux, 2010). Moreover, individual differences in language skills contribute more to text fluency than word level fluency does, and particularly for second-language or bilingual readers (Geva et al., 1997; Buly and Valencia, 2002; Geva and Farnia, 2012). While most of the children in this study rated English as their best language (probably a result of English being the main language of instruction throughout primary school), about 40% reported their mother tongue as their better language. Within this mixed group of bilinguals, neither their age of acquisition of English (by or after 3 years of age) nor their first language status (English or other language learned first) had any bearing on their text fluency performance, as indicated by between groups comparisons. But regardless of these factors, oral proficiency in English was related to their reading fluency performance, according to the correlational and regression analyses.

With regard to the contribution of text fluency to reading comprehension, it was shown that monofractal structure contributed significant variance after controlling for age, whereas multifractal structure and %Determinism did not. This contrasts with Wallot et al. (2014) wherein %Determinism was the best predictor of comprehension, while monofractal structure added unique variance only for oral and not for silent reading. Methodological differences in that study, including use of a single story matched to the youngest readers (grade 2), may explain the difference in findings. That is, while participants in Wallot et al. (2014) received the same texts and accordingly showed increases in comprehension score with age, participants in the present study received text of age-matched difficulty and accordingly did not show changes in comprehension scores with age. Alternatively, it may indicate a difference in the manner by which skilled, fluent reading emerges in monolingual vs. bilingual readers. That is, the way the reading system is assembled to perform the task of comprehending text may differ between monolingual and bilingual readers. For bilingual readers overall, the optimal state for comprehension may be a tight coupling of reading-time performance to the ongoing informational input provided by the text. Further study is required to confirm these ideas, especially given the age related differences we found.

**Figure 2:**
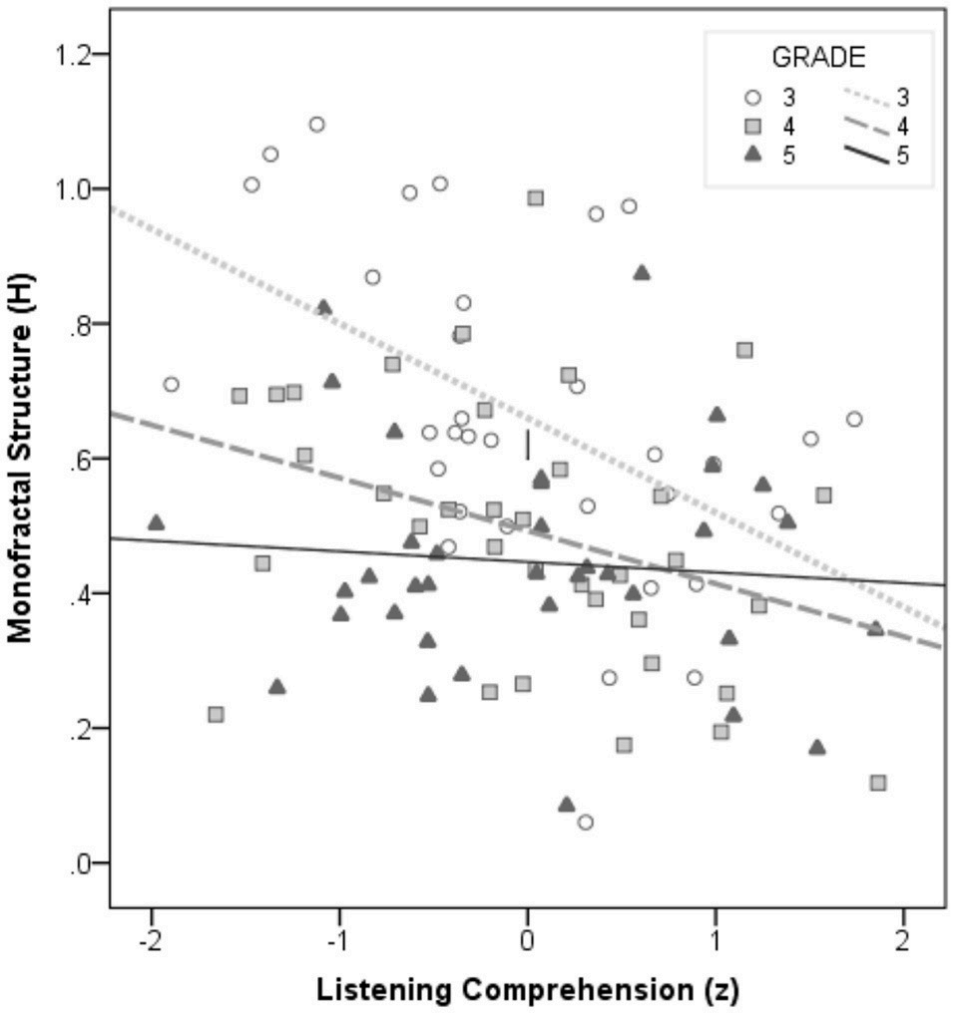
**Scatterplot of individuals' scores on English oral language proficiency and silent text fluency**. Data representing the correspondence of oral language proficiency, measured as listening comprehension (Student's t-statistic, *z*), and text reading fluency, measured as monofractal structure of reading times across the story (Hurst exponent, *H*). Individuals from grade-level groups are coded with unfilled circles (Grade 3), light squares (Grade 4), and dark triangles (Grade 5). Lines of best fit are similarly shown across the Grade 3 group (dotted line), the Grade 4 group (dashed line), and the Grade 5 group (solid line).

Nonetheless, the finding that processes for silent reading fluency, as indicated by monofractal structure here, contributes to reading comprehension coincides with earlier findings with English language learners. Oral text fluency was found to uniquely predict reading comprehension by Crosson and Lesaux (2010) with grade 5 ELLs after controlling for word reading fluency and oral language proficiency, and by Jeon (2012) with adult ELLs after controlling for word reading fluency and pseudoword reading. The present findings with bilingual children show that monofractal structure for text fluency and listening comprehension for oral language proficiency are the strongest predictors of reading comprehension of the stories. These two factors are also strongly related to each other. Their negative relation, as seen in the scatterplot (Figure 2) shows that those with stronger language proficiency also show decreased monofractal structure of their reading times (indicating increased text fluency). When broken down by age groups, we can see that this strong relation may be driven primarily by the youngest group of third graders. By grade 5, it appears language proficiency has no bearing on the text fluency metric. Further examination of the relation of both factors to reading comprehension did not support a model where text fluency mediates the relation between listening comprehension and reading comprehension (e.g., Kim and Wagner, 2015). Instead, both text fluency and listening comprehension contributed unique variance to reading comprehension when the other variable was controlled.

With regard to word level compared with passage level fluency, text fluency showed a stronger relation to reading comprehension than word fluency, though word fluency did show some correlation with comprehension despite being measured with a separate task. Of the text fluency measures, only monofractal structure showed a significant correlation and predicted unique variance in reading comprehension (8%). This is similar to the results with monolingual readers reported in Wallot et al. (2014). The relation of the monofractal structure measure and reading comprehension is negative, as found earlier, suggesting that better reading is a consequence of processes that are more strongly driven by the structure of the text. However, the corollary finding, that better reading is also characterized by cognitive reorganization during reading as indexed by a positive relation of the multifractal structure with reading comprehension, was not replicated in this sample. There was variation across the age groups, however, with regard to relations between the fluency metrics and reading comprehension. Differences across age are informative, as these were not examined previously because the sample size was smaller (Wallot et al., 2014).

For the complexity measures of silent text reading fluency, the bilingual readers showed the same pattern in %Determinism across grade level as monolingual readers of English (O'Brien et al., 2014). This measure of the degree of order in reading times across the story text showed increasing structure in reading performance with age. In the previous study, second graders showed less determinism compared with fourth and sixth graders, who in turn showed less determinism in reading times than adult readers. In the present study, the shift to greater %Determinism similarly occurred at grade 4.

We also found a difference across age groups in monofractal structure of children's reading times. Third grade children had greater monofractal exponents (*H*) compared with the older children. This differs from previous results (O'Brien et al., 2014), wherein the monolingual readers showed no age related differences in monofractal structure. In that study, one story rated as grade 2.5 (ATOS) was given to all age groups, effectively inducing greater levels of fluent reading with increasing age. Here, the reading texts were roughly matched to age groups: the 3rd grade group read a 2.4 ATOS level story, while the other groups read 4.4 and 5.3 ATOS leveled texts. While word lengths were similar across the three stories, sentences were on average longer for the 4th and 5th graders' stories. Perhaps the more complex language represented in the higher level texts allowed for, or demanded more, attention to text structure, yielding lower fractal scaling that is more constrained by faster time scales with less long-range dependencies, closer to random fluctuations that are expected to reflect a closer constraining by the text.

Age had a differential effect on multifractal scaling than it did on monofractal scaling—showing an increase in the former and a decrease in the latter across the grade level groups. This finding follows the predicted outcome that more fluent reading is related to a constraining of performance to faster time scales driven by text structure, as reflected in smaller scaling exponents in monofractal structure, whereas it is also characterized by a higher degree of adaptive changes as the reader processes the meaning of the text, as reflected in larger exponents in multifractal structure. A larger multifractal exponent indicates that, whereas reading is constrained by small timescale features of the text (e.g., word by word, or within-phrase features), the reader is also attuned to larger timescale features (e.g., in the plot or setting of the story).

Examining the correlational analyses separately for each grade, we found that the relation of comprehension to monofractal scaling was significantly negative only at grade 3, whereas the relation to multifractal scaling was positive and only significant at grade 5. %Determinism showed the same pattern as multifractal scaling, where the relation to comprehension was only significant by grade 5. The developmental differences show that these metrics for “silent fluency” may capture different aspects of what we mean by fluency—as primarily text-driven speed earlier on, but with a later emphasis on order and also adaptive aspects of fluency that contribute to better comprehension.

It should be noted that reading comprehension did not differ across the age groups, so these changes in relation to fluency aspects are not simply due to improved comprehension generally. Further, although the older readers showed both decreased monofractal and increased multifractal structure and determinism as a group, it appears that individual differences in reading comprehension were only related to the multifractal structure and determinism for the P5 group. For the P3 children, the better comprehenders looked more like the older groups, with lower monofractal structure than their peers. That is, for the older readers good comprehension may act as a dynamic attractor state (as indicated by higher %Determinism) where current processing is constrained by what has already been read, but is also responsive to how well new information is integrated with previous context (as indicated by higher multifractal structure) (e.g., Paulson, 2005). For the younger readers, on the other hand, good comprehension appears to coincide with reading activity focused at small timescales (e.g., single word level, as indicated by lower monofractal structure), and follows the concept that these readers are still at the stage where they are “glued to the print” (Chall, 1996). At this stage, the difference between better and poorer comprehenders is leveled at processing at the small time scale (e.g., word level recognition or decoding), and is not yet dependent on the readers'attunement to larger timescale features (e.g., meaning-based processing of the story). This is supported by the finding that word fluency showed a significant relation to reading comprehension only for the grade 3 group. By grade 4 and 5 it appears word fluency is no longer as important for comprehension, as text fluency becomes more prominent by grade 5 for our bilingual sample. Geva and Farnia (2012) similarly found that word level fluency only contributed to text fluency in early primary school, but by grade 5 text fluency became more aligned with language skills for both first language and second language learners.

In sum, for the bilingual readers we observed across the middle primary grades, the present results indicate that text fluency measured for silent reading predicted story reading comprehension, and that English language proficiency was also predictive of both reading fluency and reading comprehension performance. The present set of results should be treated with caution, as the sample size was small for examining predictive relations within each grade level. Further, our skills measures are based on single measurements rather than latent variables, and findings may be particular to the specific assessments we used. More research on the roles of fluency and oral language in bilingual reading is warranted, particularly given the apparent age-related variations in the relation between these skills.

## Author contributions

BO and SW designed the study. BO collected and analyzed the data. BO and SW interpreted the data and wrote the manuscript.

### Conflict of interest statement

The authors declare that the research was conducted in the absence of any commercial or financial relationships that could be construed as a potential conflict of interest.

## References

[B1] Accelerated Reader (2011). Book Finder US. Available online at: http://www.arbookfind.com/bookdetail.aspx?q=59508l=ENslid=194455065 (Accessed August 15, 2011).

[B2] Adolf S. Catts H. Little T. (2006). Should the simple view of reading include a fluency component? Read. Writ. 19, 933–958. 10.1007/s11145-006-9024-z

[B3] Baker S. K. Good R. (1995). Curriculum-based measurement of English reading with bilingual Hispanic students: a validation study with second grade students. School Psychol. Rev. 24, 561–578.

[B4] Bashir A. S. Hook P. E. (2009). Fluency: a key link between word identification and comprehension. Lang. Speech Hear. Serv. Schools 40, 196–200. 10.1044/0161-1461(2008/08-0074) 18952813

[B5] Berninger V. W. Abbott R. D. Trivedi P. Olson E. Gould L. Hiramatsu S. (2010). Applying the multiple dimensions of reading fluency to assessment and instruction. J. Psychoeduc. Assess. 28, 3–18. 10.1177/0734282909336083

[B6] Bialystok E. Luk G. Peets K. F. Yang S. (2010). Receptive vocabulary differences in monolingual and bilingual children. Bilingualism 13, 525–531. 10.1017/S1366728909990423 25750580 PMC4349351

[B7] Booth C. Brown H. L. Eason E. G. Wallot S. Kelty-Stephen D. G. (2016). Expectations on hierarchical scales of discourse: multifractality predicts both short- and long-range effects of violating gender expectations in text-reading. Discourse Process. 10.1080/0163853X.2016.1197811

[B8] Brainard D. H. (1997). The Psychophysics toolbox. Spatial Vis. 10, 433–436. 10.1163/156856897X00357 9176952

[B9] Buly M. R. Valencia S. W. (2002). Below the bar: profiles of students who fail state Reading assessments. Edu. Eval. Policy Anal. 24, 219–239. 10.3102/01623737024003219

[B10] Caccia D. C. Percival D. Cannon M. J. Raymond G. Bassingthwaighte J. B. (1997). Analyzing exact fractal time series: evaluating dispersional analysis and rescaled range methods. Phys. A 246, 609–632. 10.1016/S0378-4371(97)00363-4 22049251 PMC3205082

[B11] Chall J. S. (1996). Stages of Reading Development (2nd Edn.). Fort Worth, TX: Harcourt-Brace.

[B12] Chen X. Geva E. Schwartz M. (2012). Understanding literacy development of language minority students: an integrative approach. Read. Writ. 25, 1797–1804. 10.1007/s11145-012-9400-9

[B13] Ciuciu P. Abry P. He B. J. (2014). Interplay between functional connectivity and scale-free dynamics in intrinsic fMRI networks. Neuroimage 95, 248–263. 10.1016/j.neuroimage.2014.03.047 24675649 PMC4043862

[B14] Costa A. Roelstraete B. Hartsuiker R. J. (2006). The lexical bias effect in bilingual speech production: evidence for feedback between lexical and sublexical levels across languages. Psychon. B. Rev. 13, 972–977. 10.3758/BF03213911 17484421

[B15] Crosson A. C. Lesaux N. K. (2010). Revisiting assumptions about the relationship of fluent reading to comprehension: Spanish-speakers text-reading fluency in English. Read. Writ. 23, 475–494. 10.1007/s11145-009-9168-8

[B16] Cutting L. E. Materek A. Cole C. A. S. Levine T. M. Mahone E. M. (2009). Effects of fluency, oral language, and executive function on reading comprehension performance. Ann. Dyslexia 59, 34–54. 10.1007/s11881-009-0022-0 19396550 PMC2757040

[B17] DeRamirez R. D. Shapiro E. S. (2006). Curriculum-based measurement and the evaluation of reading skills of Spanish-speaking English language learners in bilingual education classrooms. School Psychol. Rev. 35, 356–369.

[B18] Donald M. (2007). The slow process: a hypothetical cognitive adaption for distributed cognitive networks. J. Physiol. Paris 101, 214–222. 10.1016/j.jphysparis.2007.11.006 18280714

[B19] Foorman B. R. Koon S. Petscher Y. Mitchell A. Truckenmiller A. (2015). Examining general and specific factors in the dimensionality of oral language and reading in 4^th^-10^th^ grades. J. Educ. Psychol. 107, 884–899. 10.1037/edu0000026 26346839 PMC4557887

[B20] Fuchs L. S. Fuchs D. Hosp M. K. Jenkins J. R. (2001). Text fluency as an indicator of reading competence: a theoretical, empirical, and historical analysis. Sci. Stud. Read. 5, 239–256. 10.1207/S1532799XSSR0503_3

[B21] Geva E. Farnia F. (2012). Developmental changes in the nature of language proficiency and reading fluency paint a more complex view of reading comprehension in ELL and EL. Read. Writ. 25, 1819–1845. 10.1007/s11145-011-9333-8

[B22] Geva E. Wade-Woolley L. Shany M. (1997). Development of reading efficiency in first and second language. Sci. Stud. Read. 1, 119–144. 10.1207/s1532799xssr0102_2

[B23] Geva E. Zadeh Y. Z. (2006). Reading efficiency in native English-speaking and English-as-a-second-language children: the role of oral proficiency and underlying cognitive-linguistic processes. Sci. Stud. Read. 10, 31–58. 10.1207/s1532799xssr1001_3

[B24] Geva E. (2006). Second-language oral proficiency and second-language literacy, in Developing Literacy in Second-language Learners: Report of the National Literacy Panel on Language-Minority Children and Youth, eds August D. Shanahan T. (New York, NY: Routledge), 123–140.

[B25] Goldberger A. L. Amaral L. A. Hausdorff J. M. Ivanov P. C. Peng C. K. Stanley H. E. (2002). Fractal dynamics in physiology: alterations with disease and aging. Proc. Natl. Acad. Sci. U.S.A. 99, 2466–2472. 10.1073/pnas.012579499 11875196 PMC128562

[B26] Gollan T. H. Montoya R. I. Fennema-Notestine C. Morris S. K. (2005). Bilingualism affects picture naming but not picture classification. Mem. Cogn. 33, 1220–1234. 10.3758/BF03193224 16532855

[B27] Graesser A. C. McNamara D. S. Louwerse M. M. Cai Z. (2004). Coh-metrix: analysis of text on cohesion and language. Behav. Res. Methods Instrum. Comput. 36, 193–202. 10.3758/BF03195564 15354684

[B28] Grosjean F. (2013). Bilingualism: A short introduction, in The Psycholinguistics of Bilingualism, eds Grosjean F. Li P. (Malden, MA: John Wiley & Sons), 5–26.

[B29] Holden J. G. (2005). Gauging the fractal dimension of response times from cognitive tasks, in Tutorials in Contemporary Nonlinear Methods for the Behavioral Sciences, eds Riley M. A. Van Orden G. C. 268–318. Avaialble online at: http://www.nsf.gov/sbe/bcs/pac/nmbs/nmbs.jsp

[B30] Hoover W. A. Gough P. B. (1990). The simple view of reading. Read. Writ. 2, 127–160.

[B31] Hosp M. K. Fuchs L. S. (2005). CBM as an indicator of decoding, word reading, and comprehension: do relations change with grade? School Psychol. Rev. 34, 9–26.

[B32] Ihlen E. A. F. Vereijken B. (2010). Interaction-dominant dynamics in human cognition: beyond 1/f fluctuation. J. Exp. Psychol. 139, 436–463. 10.1037/a0019098 20677894

[B33] Ihlen E. A. F. (2012). Introduction to multifractal detrended fluctuation analysis in Matlab. Front. Physiol. 3:141. 10.3389/fphys.2012.00141 22675302 PMC3366552

[B34] Jackson N. E. Lu W. H. (1992). Bilingual Precocious readers of English. Roeper Rev. 14, 115–119. 10.1080/02783199209553404

[B35] Jeon E. H. (2012). Oral reading fluency in second language reading. Read. Foreign Lang. 24, 186–208.

[B36] Jiang X. Sawaki Y. Sabatini J. (2012). Word reading efficiency, text reading fluency, and reading comprehension among Chinese learners of English. Read. Psychol. 33, 323–349. 10.1080/02702711.2010.526051

[B37] Just M. A. Carpenter P. A. Woolley J. (1982). Paradigms and processes in reading comprehension. J. Exp. Psychol. 111, 228–238. 10.1037/0096-3445.111.2.228 6213735

[B38] Kantelhardt J. W. Zschiegner S. A. Koscielny-Bunde E. Havlin S. Bunde A. Stanley H. E. (2002). Multifractal detrended fluctuation analysis of nonstationary time series. Phys. A 316, 87–114. 10.1016/S0378-4371(02)01383-3

[B39] Kelty-Stephen D. G. Palatinus K. Saltzman E. Dixon J. A. (2013). A tutorial on multifractality, cascades, and interactivity for empirical time series in ecological science. Ecol. Psychol. 25, 1–62. 10.1080/10407413.2013.753804

[B40] Kim H. J. (2014). Anomalous diffusion induced by enhancement of memory. Phys. Rev. E 90:012103. 10.1103/physreve.90.012103 25122247

[B41] Kim Y. Wagner R. K. (2015). Text (oral) reading fluency as a construct in reading development: an investigation of its mediating role for children from grades 1 to 4. Sci. Stud. Read. 19, 224–242. 10.1080/10888438.2015.1007375 25848201 PMC4384883

[B42] Kim Y. Wagner R. K. Foster E. (2011). Relations among oral reading fluency, silent reading fluency, and reading comprehension: a latent variable study of first-grade readers. Sci. Stud. Read. 15, 338–362. 10.1080/10888438.2010.493964 21747658 PMC3131673

[B43] Klauda S. L. Guthrie J. T. (2008). Relationships of three components of reading fluency to reading comprehension. J. Educ. Psychol. 100, 310–321. 10.1037/0022-0663.100.2.310

[B44] Kloos H. Van Orden G. C. (2010). Voluntary performance of cognitive and motor tasks. Mind Matter 8, 19–43.

[B45] Kuhn M. R. Schwanenflugel P. J. Meisinger E. B. Levy B. A. Rasinski T. V. (2010). Aligning theory and assessment of reading fluency: automaticity, prosody, and definitions of fluency. Read. Res. Q. 45, 230–251. 10.1598/RRQ.45.2.4

[B46] Kuznetsov N. A. Wallot S. (2011). Effects of accuracy feedback on fractal characteristics of time estimation. Front. Neurosci. 5:62. 10.3389/fnint.2011.00062 22046149 PMC3201842

[B47] Lehne M. Engel P. Rohrmeier M. Menninghaus W. Jacobs A. M. Koelsch S. (2015). Reading a suspenseful literary text activates brain areas related to social cognition and predictive inference. PLoS ONE 10:e0124550. 10.1371/journal.pone.0124550 25946306 PMC4422438

[B48] Lems K. (2006). Reading fluency and comprehension in adult English language learners, in Fluency Instruction: Research Based Best Practices, eds Rasinski T. Blachowicz C. Lems K. (New York, NY: Guilford), 231–252.

[B49] Lems K. (2012). The effect of L1 orthography on the oral reading of adult English language learners. Writ. Syst. Res. 4, 61–71. 10.1080/17586801.2011.635951

[B50] Lesaux N. K. Geva E. Koda K. Siegel L. Shanahan T. (2008). Development of literacy in second-language learners, in Developing Reading and Writing in Second-language Learners, eds August D. Shanahan T. (New York, NY: Routledge), 27–60.

[B51] Liu H. Hu Z. Guo T. Peng D. (2010). Speaking words in two languages with one brain: neural overlap and dissociation. Brain Res. 1316, 75–82. 10.1016/j.brainres.2009.12.030 20026317

[B52] Marwan N. Wessel N. Meyerfeldt U. Schirdewan A. Kurths J. (2002). Recurrence-plot-based measures of complexity and their application to heart-rate-variability data. Phys. Rev. E 66:026702. 10.1103/physreve.66.026702 12241313

[B53] Marwan N. (2011). Commandline Recurrence Plots. Available online at: http://tocsy.pik-potsdam.de/commandline-rp.php (Accessed on August 16, 2011).

[B54] McBride-Chang C. (2004). Children's Literacy Development, Texts in Developmental Psychology Series. London: Oxford Press.

[B55] Nelson G. (2002). The Singapore Corpus. International Corpus of English. Available online at: http://ice-corpora.net/ice/icesin.htm

[B56] O'Brien B. A. Wallot S. (2014). Dynamical structure of silent reading fluency in bilingual students, in Proceedings of the XVIth European Conference on Developmental Psychology, (Bologna: Medimondo), 175–179.

[B57] O'Brien B. A. Wallot S. Haussmann A. Kloos H. (2014). Using complexity metrics to assess silent reading fluency: a cross-sectional study comparing oral and silent reading. Sci. Stud. Read. 18, 235–254.

[B58] Oller D. K. Pearson B. Z. Cobo-Lewis A. B. (2007). Profile effects in early bilingual language and literacy. Appl. Psycholinguist. 28, 191–230. 10.1017/S0142716407070117 22639477 PMC3358777

[B59] Paulson E. J. (2005). Viewing eye movements during reading through the lens of chaos theory: how reading is like the weather. Read. Res. Q. 40, 338–358. 10.1598/RRQ.40.3.3

[B60] Peng C. K. Havlin S. Stanley H. E. Goldberger A. L. (1995). Quantification of scaling exponents and crossover phenomena in nonstationary heartbeat time- series. Chaos 5, 82–87. 10.1063/1.166141 11538314

[B61] Pikulski J. J. Chard D. J. (2005). Fluency: bridge between decoding and reading comprehension. Read Teacher 58, 510–519. 10.1598/RT.58.6.2

[B62] Piper B. Schroeder L. Trudell B. (2016). Oral reading fluency and comprehension in Kenya: reading acquisition in a multilingual environment. J. Res. Read. 39, 133–152. 10.1111/1467-9817.12052

[B63] Proctor C. P. Carlo M. August D. Snow C. (2005). Native Spanish-speaking children reading in English: toward a model of comprehension. J. Educ. Psychol. 97, 246–256. 10.1037/0022-0663.97.2.246

[B64] Rosseel Y. (2012). Lavaan: an R package for structural equation modeling. J. Stat. Softw. 48, 1–36. 10.18637/jss.v048.i02

[B65] Samuels S. J. (2006). Toward a model of reading fluency, in What Research has to Say About Fluency Instruction, eds Samuels S. Farstrup A. (Newark, DE: International Reading Association), 24–46.

[B66] Sandoval T. C. Gollan T. H. Ferreira V. S. Salmon D. P. (2010). What causes the bilingual disadvantage in verbal fluency? The dual-task analogy. Biling Lang. Cogn. 13, 231–252. 10.1017/S1366728909990514

[B67] Schatschneider C. Fletcher J. M. Francis D. J. Carlson C. D. Foorman B. R. (2004). Kindergarten prediction of reading skills: a longitudinal comparative analysis. J. Educ. Psychol. 96, 265–282. 10.1037/0022-0663.96.2.265

[B68] Schilling H. E. Rayner K. Chumbley J. I. (1998). Comparing naming, lexical decision, and eye fixation times: word frequency effects and individual differences. Mem. Cogn. 26, 1270–1281. 10.3758/BF03201199 9847550

[B69] Segalowitz N. Hulstijn J. (2005). Automaticity in bilingualism and second language learning, in Handbook of Bilingualism: Psycholinguistic Approaches, eds Kroll J. F. De Groot A. M. B. (Oxford: Oxford University Press), 371–388.

[B70] Silverman R. D. Speece D. L. Harring J. R. Ritchey K. D. (2013). Fluency has a role in the simple view of reading. Sci. Stud. Read. 17, 108–133. 10.1080/10888438.2011.618153

[B71] Stephen D. G. Boncoddo R. A. Magnuson J. S. Dixon J. A. (2009). The dynamics of insight: mathematical discovery as a phase transition. Mem. Cogn. 37, 1132–1149. 10.3758/MC.37.8.1132 19933457

[B72] Torgesen J. K. Wagner R. K. Rashotte C. A. (1999). Test of Word Reading Efficiency. Austin, TX: PRO-ED.

[B73] Van Heuven W. J. Dijkstra T. Grainger J. (1998). Orthographic neighborhood effects in bilingual word recognition. J. Mem. Lang. 39, 458–483. 10.1006/jmla.1998.2584

[B74] Van Orden G. Kloos H. Wallot S. (2011). Living in the pink: intentionality, wellness, and complexity, in Handbook of the Philosophy of Science, Vol. 10, Philosophy of Complex Systems, ed Hooker C. A. (Amsterdam: Elsevier), 639–684. 10.1016/b978-0-444-52076-0.50022-5

[B75] Verhoeven L. van Leeuwe J. (2012). The simple view of second language reading throughout the primary grades. Read. Writ. 25, 1805–1818. 10.1007/s11145-011-9346-3 22923881 PMC3422459

[B76] Wagner R. K. Torgesen J. K. Rashotte C. A. (1999). Comprehensive Test of Phonological Processing. Austin, TX: PRO-ED.

[B77] Wallot S. Fusaroli R. Tylén K. Jegindø E. M. (2013). Using complexity metrics with RR intervals and BPM heart rate measures. Front. Physiol. 4:211 10.3389/fphys.2013.00211 PMC3741573 23964244

[B78] Wallot S. O'Brien B. A. Haussmann A. Kloos H. Lyby M. S. (2014). The role of reading time complexity and reading speed in text comprehension. J. Exp. Psychol. 40, 1745–1765. 10.1037/xlm0000030 24999710

[B79] Wallot S. O'Brien B. Coey C. A. Kelty-Stephen D. (2015). Power-law fluctuations in eye movements predict text comprehension during connected text reading, in Proceedings of the 37th Annual Meeting of the Cognitive Science Society, eds Noelle D. C. Dale R. Warlaumont A. S. Yoshimi J. Matlock T. Jennings C. D. (Austin, TX: Cognitive Science Society), 2583–2588.

[B80] Wallot S. O'Brien B. A. Van Orden G. (2012). Fractal and recurrence analysis of psycholinguistic data, in Methodological and Analytic Frontiers in Lexical Research, eds Westbury C. Jarema G. Libben G. (Amsterdam: John Benjamins), 395–430. 10.1075/bct.47.18wal

[B81] Wallot S. (2015). From “cracking the orthographic code” to “playing with language”: toward a usage-based foundation of the reading process. Front. Psychol. 5:891 10.3389/fpsyg.2014.00891 PMC4141234 25202285

[B82] Wallot S. (2016). Understanding reading as a form of language-use: a language game hypothesis. New Ideas Psychol. 42, 21–28. 10.1016/j.newideapsych.2015.07.006

[B83] Webber C. L. Zbilut J. P. (1994). Dynamical assessment of physiological systems and states using recurrence plot strategies. J. Appl. Physiol. 76, 965–973. 8175612 10.1152/jappl.1994.76.2.965

[B84] Webber C. L. Zbilut J. P. (2005). Recurrence quantification analysis of nonlinear dynamical systems, in Tutorials in Contemporary Nonlinear Methods for the Behavioral Sciences, eds Riley M. A. Van Orden G. C. 26–96. Available online at: http://www.nsf.gov/sbe/bcs/pac/nmbs/nmbs.jep (Accessed on August 23, 2009).

[B85] Wijnants M. L. Bosman A. M. T. Hasselman F. Cox R. F. A. Van Orden G. (2009). 1/f scaling in movement time changes with practice in precision aiming. Nonlinear Dyn. Psychol. Life Sci. 13, 79–98. 19061546

[B86] Wijnants M. L. Hasselman F. Cox R. F. A. Bosman A. M. T. Van Orden G. (2012). An interaction-dominant perspective on reading fluency and dyslexia. Ann. Dyslexia 62, 100–119. 10.1007/s11881-012-0067-3 22460607 PMC3360848

[B87] Wolf M. Denckla M. B. (2004). Rapid Automatized Naming and Rapid Alternating Stimulus tests (RAN/RAS). Austin, TX: PRO-ED.

[B88] Woodcock R. W. McGrew K. S. Mather N. (2007). Woodcock-Johnson III Tests of Achievement with Normative Update. Rolling Meadows, IL: Riverside Publishing.

[B89] Zeno S. M. Ivens S. H. Millard R. T. Duvvuri R. (1995). The Educator's Word Frequency Guide. Brewster, NY: Touchstone.

