# Supplementary material for: Silent Reading Fluency and Comprehension in Bilingual Children
Source: Front Psychol. 2016 Aug 31;7:1265. doi: 10.3389/fpsyg.2016.01265 (PMC5005424; doi:10.3389/fpsyg.2016.01265)
Supplement: Supplementary file 1 [file DataSheet1.docx]

**Appendix**

This appendix gives a brief overview over how the complexity measures used in this study – Recurrence Quantification Analysis (RQA), Detrended Fluctuation Analysis (DFA), and Multifractal Detrended Fluctuations Analysis (MFDFA) – are computed (see also Wallot et al., 2014). Moreover, we will summarize briefly how these measures have been interpreted in the context of psychological research in general, and of reading/language production and perception in particular.

**Recurrence Quantification Analysis (RQA) for the estimation of deterministic structure (%Determinism) in a time-series**

The central concept of Recurrence Quantification Analysis (RQA) is repetition, and the analysis quantifies various aspects of patterns in a time-series repeat (Webber & Zbilut, 1994). RQA actually delivers many different outcome measures that describe the dynamics of a time-series (Marwan, Wessel, Meyerfeldt, Schirdewan, & Kurths, 2002). In the present study, we were interested in the RQA measure of %Determinism (percent determinism), which quantifies the degree of predictability in a time-series, and the extent to which values in the time-series repeat in larger patterns. In order to calculate RQA measures such as %Determinism for a time-series, the recurrence plot (RP) of that time-series needs to be derived, which is the basis for the calculation of any recurrence measure. In order to construct a proper RP, one needs to use the method of time-delayed embedding, whereby a one-dimensional time-series is plotted against itself at a certain lag to construct a phase-space profile of that time-series. To embed a time-series properly (i.e., to figure out how often the time-series has to be plotted against itself and at which delay), several parameters have to be estimated (see Webber & Zbilut, 2005).

The first parameter is the delay parameter tau (τ). Estimation of τ is especially important for continuously sampled data. This can be done by calculating the Average Mutual Information function for over several lags of the time-series, and then picking the first local minimum of mutual average information as an estimate of the delay parameter. However, in our present study we recorded time-series of key-press intervals from self-paced reading, which are series of inter-event-times, in which case delaying of the time-series is usually necessary, and the delay parameter τ can simply be set to 1 (Charles Webber, personal communication).

The second parameter is the embedding dimension (D), and corresponds to the number of times one needs to plot the time-series against itself, in order to create a proper phase-space profile. This can be done using the False-Nearest Neighbor (FNN) function. The basic idea is that if the phase-space of a time-series is too small, then data points in that phase-space lie close together (i.e., are neighbors) just because the time-series has not been embedded in a large-enough space. This can be solved by seeking an embedding dimension *D* where data-points are sufficiently far away from each other and the number of neighbors stays relatively constant. Similarly as with finding the delay parameter using Average Mutual Information, one embeds the time-series into consecutively higher dimensions, and observes how the number of false neighbors changes with increases in dimensionality. Again, the first local minimum of that function serves as an estimate for the embedding dimension *D*.

After the estimation of τ and *D*, the time-series *x* can be embedded into a phase-space (see Equation 1):

 (1)

where

*v_i_* = is the *D*-dimensional vector that constitutes the phase-space.

*X_i_* = is the *i^th^* sub-series of the time-series *x* that is used for embedding.

τ = the delay parameter that gives the lag at which the time-series *x* is embedded.

*D* = is the dimensionality of the reconstructed phase-space.

In order to make different data sets comparable, one needs to normalize the resulting phase-space for each individual time-series. This is done by selecting a norm parameter. Normalization of the phase-spaces ensures that differences observed between data sets are actually a function of the sequential ordering in a time-series, and not simply due to differences in the overall magnitude of the values between different time-series. Various norms can be applied, but for our present study we chose the Euclidean norm (i.e., the average distance between all points in phase-space for a particular time-series), by which the coordinates in each individual phase-space were divided.

Finally, one needs to select a radius parameter, within which the different coordinates in phase-space are either counted as being recurrent (i.e., all points lying within the selected radius are counted as recurrent) or not. There are usually no absolute values for the radius parameter in psychological data sets. Rather, it is recommended that the radius is set in a way that 1-5% of the data points in a time-series are counted as recurrent (Webber & Zbilut, 2005). However, for inter-event times such as our data, the percentage of recurrent points can also be higher than that (Wallot, O’Brien & Van Orden, 2012).

The basic measure for all other RQA variables is %Recurrence, which is simply the sum of all recurrent points in an RP (i.e., all points that are closer to each other than the radius parameter in phase-space) divided by the size of the plot (see Equation 2):

 (2)

From %Recurrence, we can now compute % Determinism as the number of all recurrent points that are diagonally adjacent to each other divided by the sum of all recurrent points on RP. In the context of our present study, %Determinism is a measure of how much reading times are organized in larger temporal patterns that spanning reading of multiple words times (see Equation 3):

 (3)

**Interpretation of RQA %Determinism**

First studies using complexity metrics as descriptors of the reading process in oral and silent reading found %Determinism of reading times to be predictive of reader skill (O’Brien et al., 2014) and comprehension (Wallot et al., 2014). In particular, the higher %Determinism of reading times was, the better readers comprehended a text and the faster their reading speed. That is, the more temporally structured their reading times, the better they were. The question is, where did the temporal structure in reading times come from? Most models of reading assume that specific lexical or syntactic factors exhibit specific effects on the local reading process (e.g., high word frequency of a word leads to shorter fixation durations, Schilling et al., 1998). However, individual text features, such a word frequency, do not play much of a role in naturalistic text reading (Wallot et al., 2012).

Based on these set of findings, Wallot (2015; 2016) formulated the language game hypothesis of reading (LHG), according to which the informational structure of a text constrains the reading process, and if a reader possesses the necessary reading capacities, these constraints help to coordinate the cognitive processes during reading in a way that is optimal, given an individual reader’s capacity and reading history. This way, one need not assume that specific text features always serve the same specific role for all readers, but that readers use whatever informational structure the texts provides for them to guide their reading successfully.

One prediction from LGH is that the better readers are able to harness the informational structure of a text, the more their cognitive processes are constrained towards focusing on the relevant informational features of a text, and the more fluent and effortless their reading is. In this context, %Determinism can be seen as a general measure of the degree of constraint a reading task puts on the coordination of cognitive processes, and high values of %Determinism in measures of the reading process should be positively correlated with reading outcomes, such as speed and comprehension. Beyond these psychological considerations, there is also a statistical analogy between %Determinism and the notion of fluency: If we measure the dynamics of some physical system, for example water running down a river, then %Determinism is correlated with flow characteristics of that system, meaning that the more smooth and undisturbed the flow is, the higher its measured %Determinism. This statistical notion of flow coincides with the concept of fluid reading in psychology, and LGH provides a common framework for these concepts.

**Detrended Fluctuation Analysis (DFA) for the estimation of monofractal fluctuations in a time-series**

**The DFA procedure**

Detrended fluctuation analysis (DFA) estimated monofractal scaling (also referred to as long-range-correlations or long-memory) of a time-series (Peng et al., 1995). The principle idea of DFA is to examine how variance in a time-series changes as a function of window size. The change in variance with window size is related to a specific form of autocorrelation in the time-series, called monofractal scaling or long-memory (Kelty-Stephen & Wallot, 2016). The term long-memory is perhaps more intelligible, as it contrasts with ‘short-memory’ in a time-series: While short-memory implies that there are only limited autocorrelation processes in a time-series (e.g., significant correlation at lag1, lag2, and lag3), long-memory implies that the autocorrelation never does completely drop to 0, and hence significant autocorrelation exists across (almost) all lags of the time-series. Now, DFA estimates the strength of such long-memory in a time-series, and summarizes it in a single value, the so-called Hurst-exponent *H*. The bigger *H*, the stronger long-memory, a.k.a. monofractal scaling. Conventionally, *H* = 0.5 indicates the absence of long memory (i.e., white noise), while *H* > .05 indicates persistent long-memory (positive autocorrelation), and *H* < 0.5 indicates anti-persistent long-memory (negative autocorrelation).

To compute *H*, DFA performs the following steps: If the time-series is of the fGn-type (fractional Gaussian noise – our self-paced reading data is of this type), then the time-series needs to be integrated first. If the time-series is of the fBm-type (fractional Gaussian motion), conventionally no integration is performed. Next, as we are interested in how variance changes across different window sizes in the time-series, the first step in DFA is to break the time-series into (preferably non-overlapping) windows of different size. Usually, the smallest window size consists of 4 adjacent data point, and the largest window size is equal to length of the time-series divided by 4. One of our stories contained 1105 words. When we divide this by 4, we are left with four sub-windows of the time-series containing 276 data points each, with the last two 2 data points being lost for the analysis at that window size. If a time-series is not neatly dividable by 4, the loss of the remaining data points usually does not impact the results of DFA, but re-sampling techniques can be used to avoid such losses (Kantelhardt, Zschiegner, Koscielny-Bunde, Bunde, Havlin, & Stanley, 2002).

Since simple long-term trends might be present in a time-series, which are not part of the autocorrelation structure, potential trends need to be removed, as they can bias the estimation of *H* (Caccia, Percival, Cannon, Raymond, & Bassingthwaighte, 1997). So after dividing a time-series into subsets of equal window size, each sub-series is individually detrended – hence the name of the analysis. Usually this is done by removing linear trends, but removing quadratic or cubic trends is also possible. For our present data, we choose simple linear detrending. After linear trends have been removed from the data in each sub-window, the root-mean-square (*RMS*) for each sub-series is calculated across all sub-windows and averaged to result in the average fluctuation magnitude for that window size (*RMS_s_*), see Equation 4:

 (4)

where

*RMS_s_* = root-mean-square for a particular scale of window size.

*N* = number of sub-series at a particular window size.

*y(k)* = integrated sub-series *k*.

*y_s_(k)* = local trend for at a particular bin size for the particular sub-series *k* of *y*.

As we are interested in how fluctuation magnitude (variance) changes with window size, we now need to apply this procedure systematically across many window sizes, from the smallest (in our case 4) to the biggest (in our case a quarter of the length of a time-series). Hence, starting from the smallest window size, this process is repeated for increasingly larger bin sizes until reading the maximum window size. After having performed this calculation for across several window-sizes, from the smallest to the largest, the results are plotted on a log-log scale, i.e., the logarithm of window size from smallest to largest on the x-axis and the logarithm of the *RMS* on the y-axis. If the logarithm of window-size scales linearly with logarithm of *RMS*, a trend line is fitted, and the slope of that line (*S)* estimates the strength of monofractal scaling, *H*, where *H* = *S*.

**Interpretation of *H***

Similarly to RQA %Determinism, it has been found that decreased *H* of time-series of reading time data was positively correlated with aspects of reading skill (O’Brien & Wallot, 2014; O’Brien et al. ,2014; Wallot et al., 2014; Wallot, O’Brien, Coey, & Kelty-Stephen, 2015). However, as we explained, *H* is not a measure of the degree of temporal structure in time-series data in the sense that %Determinism is, but rather it quantifies the strength of long-memory processes in the time-series, i.e., how interdependent different data points are. In (neuro)physiology, this interdependence has been though to reflect the (optimal) coordination of organ systems (Goldberger et al., 2002) or the degree of connectivity in the brain (Ciuciu et al., 2014), and evidence of strong long-memory seemed to capture optimal endogenous (neuro-)physiological activity in the absence of any particular environmental influence on the organism (Van Orden et al., 2011).

However, when an organism is performing a physical task, then endogenous activity of the (peripheral) physiology re-organizes to fit the physical task demands (Wallot et al., 2013), or in the case of neuro-physiological activity, the cognitive task demands. Using a simple tapping study, Kuznetsov and Wallot (2011) showed that endogenous tapping active during time-estimation exhibited strong traces of long-memory (i.e., high *H*), reflecting endogenous motor-cognitive activity, relatively unconstrained by environmental information that was task relevant. However, when providing participants with visual feedback about their task performance, that is increasing the coupling of participants’ time-estimates to an external source of information, long-memory as an indicator of endogenous motor-cognitive dynamics decreased, as the information provided by external the visual feedback was increased. Relating the strength of long-memory in time-series of cognitive performance to sources of internal (endogenous) control vs. external, environmentally driven control, Kloos and Van Orden (2010; Van Orden et al., 2011) hypothesized that the degree to which long-memory strength, i.e., values of *H*, decreases, this is indicative of the degree of sources of external control taking over performance of a cognitive task and interfering with the ‘natural’, endogenous motor-cognitive dynamics.

Hence, in the absence of any active task performance contingent on environmental information, behavioral measures of cognitive performance that could be used to study reading (eye-movements, finger movements during key-press collection) would reveal a high degree of endogenous fluctuations as indicated by monofractal scaling (i.e., high *H*). Moreover, the degree to which environmental, task-relevant information co-controls motor-cognitive activity (such as the print on a page during reading, upon which eye-movements and key-presses become contingent) will reveal itself in decreased monofractal structure (i.e., low *H*), in the measurement in question. In conjunction with LGH (Wallot, 2015; 2016), this implies that the stronger a reader’s reading process is controlled by the relevant informational characteristics of a text, the lower monofractal structure (i.e., *H*) should be, and putatively the better off a reader she is, as her cognitive performance is optimally co-controlled by the relevant information structure of the (reading) task at hand (Kloos & Van Orden, 2010; Van Orden et al., 2010). Coincidently, also *H* is a measure of flow-diffusion characteristics in physical systems, where *H* = 0.5 is associated with homogenous diffusion (in analogy to concepts of a smooth, unperturbed reading process) and *H* > 0.5 is associated with so-called anomalous diffusion (Kim, 2014).

**Multifractal Detrended Fluctuation analysis (MFDFA) for the estimation of multifractal fluctuations in a time-series**

**The MFDFA procedure**

Multifractal Detrended Fluctuation Analysis (MFDFA) is an extension of DFA, as the name implies. While DFA tests for the presence of a single fractal or long-memory relationship in a time-series (*mono*fractal), MFDFA tests for multiple fractal relationship (*multi*fractal). MFDFA is warranted when *H* is not constant across a time-series set, but when *H* is varying significantly as a function of time. For example, this is often evident in changes between persistent and anti-persistent fluctuations in the time series, or bursting behavior. In order to quantify whether there are systematic variations of *H* in a time-series, the so-called multifractal spectrum (*MF*) is calculated. Effectively, *MF* provides the range of variation of different values of *H* observed in a single time-series.

The calculation of *MF* can be done by a so-called direct estimation (Ihlen, 2012), where a series of local Hurst exponents *H* are calculated within a single time-series, and the range of the distribution of local Hurst exponents estimates *MF*. However, one can also use an indirect estimation of MF, which we used for our data (see also Ihlen, 2012). Instead of calculating H for many sub-windows of a time-series, as done by the direct estimation method, the indirect estimation of MF proceeds basically in the same way as the regular DFA. The advantage of using the indirect method is, that one can still incorporate data from the largest window sizes in a time-series, and the spread in H is not just based on small sub-sets of that time-series.

The key difference between simple DFA and the indirect estimation of *MF* using MFDFA is, that in addition to computing *H* just for the standard *RMS*, one also computes *H* exponents for different *q*^th^-orders of *RMS* (see Equation 5): While the standard *RMS* computes the average of the squared values (i.e., *q* = 2), the *q*-order *RMS* also average across a range of larger and smaller exponents *q*. In addition to estimated H based on the fluctuation function for the standard *RMS* with *q* = 2, we computed additional *H* exponents for a range of *q*-values from 0.1 to *q* = 3.0 (see Ihlen & Verejken, 2010, for choosing the range of q-values for data of the response-time type):

 (5)

where

*RMS_sq_* = root-mean-square for a particular scale for a particular *q*-order.

*N* = number of sub-series at a particular bin size.

*y(k)* = integrated sub-series *k*.

*y_s_(k)* = local trend for at a particular bin size for the particular sub-series *k* of *y*.

*q* = the exponent of fluctuation magnitude.

The different *q*-order *RMS*’ effectively accentuate the magnitude of fluctuations on different scales, where small *q* accentuate the fluctuations on faster scales (i.e., smaller window-sizes), and larger *q* accentuate the fluctuations on slower scales (i.e., bigger window-sizes). If a time-series is composed of homogenous long-range correlations, then *H* will not change with variations in *q*. However, if long-range correlations in a time-series are changing, then *H* will be different for smaller and bigger *q*, and the range of *H* across different values of *q* (*Hq*_max_ - *Hq*_min_) can be used to estimate the multifractal spectrum *MF* (for further details on the computation of the multifractal spectrum, as well as a good introductory tutorial for multifractal analysis, see Kelty-Stephen, Palatinus, Saltzman, and Dixon, 2013).

**Interpretation of *MF***

Again, one prior study found a positive relationship between the size of MF in reading times, and subsequent text comprehension of readers (Wallot et al., 2014). What is missing in the picture about optimally coordinated reading processes in the above presented measures of %Determinism and *H* is a more flexible, adaptive element: Both measures seem to capture reading fluency in terms of its stability, i.e., a reader proceeding smoothly through a text, without greater hurdles and perturbation. However, texts and stories are not that homogenous, and the quality and engaging natures of many – if not most – stories relies on plot-twists, suspense and surprise (Lehne et al., 2015), which seem at odds with a picture of the reading process that only emphasizes a steady, smooth progress. Rather, readers must also be able to adapt to singular, crucial pieces of information of a text that are of outstanding importance and can greatly change the interpretation of what has been read before. However, with the premise of skilled reading being a more efficient and effortless process than unskilled reading, it seems that skilled readers should be more quick in adapting to the introduction of new pieces of information of outstanding importance in a text, changing their understanding of the text appropriately, and settling quickly back into the smooth, effortless, and fluent mode of reading.

Other studies investigating the emergence of new representations in the learning process of mathematical problem solving found that changing structure in the participants’ performance was predictive of participants achieving a new qualitative understanding of a mathematical problem, and successfully integrating this new knowledge for further task performance (Stephen et al., 2009). Hence, Wallot and colleagues (2014) interpreted the presence of MF in reading times as an indicator that the readers successfully master integration of particular important pieces of information during reading and adapt their understanding accordingly, to proceed further with their reading in a smooth and fluent manner. Indeed, recent investigations of the reading process during text reading of a story that contained an unexpected surprising event show that MF of reading times predicted how much readers were affected by the surprising event, and readers with high MF settled back on faster reading speed after the surprising event (Booth et al., 2016). Hence, high *MF* in measures of the reading process seem to be a correlate of the ability of a reader to flexibly adapt to more complex informational change in a text during reading.
